# Ants Can Expect the Time of an Event on Basis of Previous Experiences

**DOI:** 10.1155/2016/9473128

**Published:** 2016-06-14

**Authors:** Marie-Claire Cammaerts, Roger Cammaerts

**Affiliations:** Département de Biologie des Organismes, Faculté des Sciences, Université Libre de Bruxelles, CP 160/12, 50 avenue F. D. Roosevelt, 1050 Bruxelles, Belgium

## Abstract

Working on three ant species of the genus* Myrmica*,* M. ruginodis*,* M. rubra*, and* M. sabuleti*, we showed that foragers can expect the subsequent time at which food will be available on the basis of the previous times at which food was present. The ants acquired this expectative ability right after having experienced two time shifts of food delivery. Moreover, the ants' learning score appeared to be a logarithmic function of time (i.e., of the number of training days). This ability to expect subsequent times at which an event will occur may be an advantageous ethological trait.

## 1. Introduction

Many animals can memorize the places where an event occurs. For example, using feeders, Laca [1] experimentally showed that steers (*Bos taurus x B. indicus*) avoid areas where they learned that no food is available and present long-term spatial memory for returning to previous food loaded locations. Ksiksi and Laca [2] moreover demonstrated that steers remembered food locations for at least 48 days. Working with feeders in a seminatural environment experiment, Winter and Stich [3] showed that nectar-feeding bats (*Glossophaga soricina*) learn to avoid depleted food locations and are able to memorize 40 behavior actions for efficiently finding food. More commonly, everyone can observe such ability in crows, cats, or foxes, among others.

Animals can also learn the time of the day at which an event regularly occurs and may then present some anticipation. For instance, bees, dogs, and cats can react to the presence of food some time before its effective delivery [4]. Several species can even learn both the time and the place at which an event regularly occurs; that is, they are able to acquire spatiotemporal learning. Cats, dogs, birds, and foxes among others visit places where food is commonly available at the times at which food is susceptible to be present (observations anyone can do). Working on ants, we demonstrated that also these insects can acquire spatiotemporal learning [5] and that young ants do not yet exhibit this ability [6].

In the wild, an event may not always occur at the same place, but often at different locations. This is the case for food progressively consumed (crops, flower nectars, fruit, and prey) which therefore becomes located farther or more aside. Are animals able to adapt themselves to such food relocation? We have already examined the ability to expect the place of a subsequent potential food location in the ant species* Myrmica ruginodis* Nylander, 1846, and found that workers of this species acquire it after two food shift training episodes (paper accepted for publication). We also showed that* M. sabuleti* Meinert, 1861, workers possess this ability and that it is not detained by callow ants but is acquired in the course of their experiences [7].

An event may also occur at the same place, but at different times. For instance, nectar availability or presence of insect prey depends on the light intensity and thus on the time of the day, which changes in the course of the seasons, due to the progressive increase or decrease of the daily lit period. The ability to guess the time of the occurrence of an event, for example, the presence of food on the basis of the time at which this event previously occurred, would be advantageous for an animal. It could then be ready and fully efficient at the time at which the event is expected to occur and would not lose time and energy before and after the occurrence of the event.

We wanted to know if ants are able to present some expectative behavior as for a progressive change in the time of occurrence of an event. In other words, can ants perceive that an event occurs progressively more later and act consequently?

This presumption is not nonsense because ants can acquire temporal learning [8] and spatiotemporal learning [5] and have a notion of the running time [9] (they can learn that an event lasts 5, 10, or 15 min). They also possess the four ethological abilities required for presenting expectative behavior: knowing the areas where food is usually available, having some rather long-lasting memory, being rather provident, and presenting some anticipative behavior. This has been observed in the course of our own studies as well as by other researchers [10, 11]. Ants duly mark their foraging area and memorize visual as well as olfactory cues for navigating. They have a rather long lasting memory. Some species make seed provisions; other ones have workers devoted to stock honey in their gaster. Ants can also react before the occurrence of an event [5]. It is thus possible that ants have the ability we aim to examine, that of expecting the occurrence time of an event when that time progressively differs each day, that is, the ability of expecting an event on the basis of its previous occurrence times.

We opted to work on the ant* M. ruginodis*, the biology of which we know rather well [10]. We have already studied its eye morphology, subtended angle of vision, visual perception (the workers can distinguish different patterns of small luminous spots located above them), navigation system (the species uses visual cues and uses odors only when visual cues are absent), visual and olfactory conditioning (*M. ruginodis* has a long lasting visual memory), and recruitment strategy. As stated above,* M. ruginodis *workers do have some expectative behavior as for the exact place at which an event occurs (paper accepted for publication).

In this work, we aimed to go a step further by analyzing if ants of this species can expect the exact time at which an event will occur on the basis of the previous times at which this event occurred. After the end of this experimental work, we checked our results by making similar experimentation on two other species:* M. rubra* Linnaeus, 1758, and* M. sabuleti*, the biology of which we know as well as that of* M. ruginodis* [10]. Briefly,* M. rubra *has a visual perception of medium quality and uses visual and/or olfactory cues for navigating depending on the light intensity, while* Myrmica sabuleti *has a visual perception of poor quality and essentially uses odors for navigating.

## 2. Material and Methods

### 2.1. Collection and Maintenance of Ants

The experiment was performed on four colonies of* M. ruginodis* collected in an abandoned quarry of the Aise Valley (Ardenne, Belgium), on the borders of a forest, the ants nesting under stones or in wood.* Myrmica ruginodis *is a trans-Palearctic ubiquist species largely present in moist and cool sheltered environments. It nests under stones, in rotten wood or litter, in colonies up to a thousand of workers. The workers eat small invertebrates as well as sugared food such as aphid honeydew or floral nectar. The collected colonies were demographically similar, each containing a queen, brood, and about 500 workers.

The checking experiment was made on two small colonies of* M. rubra *and two of* M. sabuleti. Myrmica rubra *is another trans-Palearctic and ubiquist ant, having a similar way of life to* M. ruginodis*, but it lives in open environments. Its colonies were collected in the Aise Valley on grassland.* Myrmica sabuleti *is a European ant living in warm and dry environments. One colony was collected in the Aise Valley on an area covered with grass and odorous plants, the other at Audregnies, on an abandoned coal-mining heap (Terril de Ferrand, Hainaut, Belgium). These small colonies contained about 150 workers and brood.

All the colonies were maintained in the laboratory in artificial nests made of one to three glass tubes half-filled with water, with a cotton-plug separating the ants from the water. The glass tubes were deposited in trays (34 cm × 23 cm × 4 cm), the internal sides of which were slightly covered with talc to prevent ants from escaping. These trays served as foraging areas, food being delivered in them. The ants were fed with a 30% saccharose aqueous solution provided* ad libitum* in a small tube plugged with cotton, each tube containing 5 mL of solution, and with two cut* Tenebrio molitor *Linnaeus, 1758 larvae provided three times a week on a glass slide. These mealworms were reared and, as the sugar water, were prepared as food in a room distinct from that where the experiments were performed. The ants could thus not see nor olfactorily perceive the food before they received it. During the experiment, food delivery was planned in time and space as detailed below. Temperature was between 18° and 22°C and relative humidity was about 80%. Lighting had a constant intensity of 330 lux while caring for the ants and testing them; during other time periods, it was dimmed to 110 lux, an intensity under which the ants could still see their environment [12]. The ambient electromagnetic field had an intensity of 2-3 *μ*W/m^2^. All the members of a colony are herein named nestmates, as commonly done by researchers on social hymenoptera.

### 2.2. Experimental Protocol

**Figure 1:**
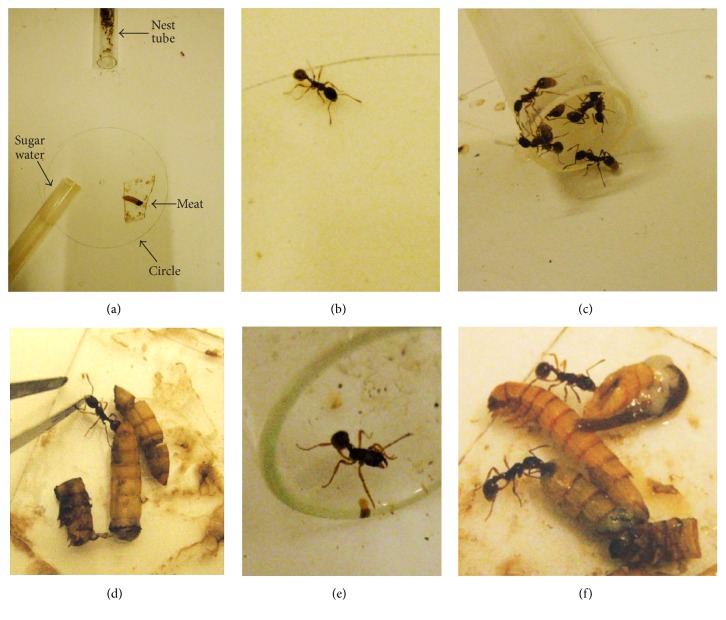
Some views of the experimental work. ((a)–(d))* Myrmica ruginodis*; ((e) and (f))* M. sabuleti*. (a) Experimental design, with a pencil drawn faint circle delimiting the food site where meat and sugar water were delivered each day at a different time. (b) Getting over the circle, an ant waiting for food a few minutes before the subsequent delivery time. (c) Ants drinking sugar water as much as they could, since they progressively learned that this food will be soon retrieved. (d) An ant gripping the entomological forceps when the observer had to remove the meat food, the ant tempting to go on eating. (e) An ant waiting for the expected food delivery from its nest entrance. (f) Ants eating meat without stopping, during the short food delivery period.

Four days before starting the experiment, the ants' food was removed from the trays which were prepared for experimenting. In the middle of the area lying in front of the nest entrances, a circle (*R* = 4 cm) was lightly pencil-drawn, defining the feeding place (the food site) on which food will be delivered and where ants will be counted during given time periods (Figure 1(a)). A first control counting was performed without food, ants being counted 10 times during a five-minute interval at *t*1 = 14:00 ± 2.5 min and the obtained numbers of ants added. The mean of the sum for the four colonies was established. In the same way, the ants were counted on the feeding place at *t*1 + 20 min, *t*1 + 40 min, *t*1 + 60 min, and *t*1 + 80 min (Tables 1 and 2). After that, food was delivered to the ants on the delimited food site. Three days before starting the experiment, a second control counting was performed in the same way, but in presence of this one-day old food (Tables 1 and 2).

At the experimental day 1, the four-day-old food left during the first control was removed two hours before the start of the experiment and ants were counted ten times at *t*1 = 14:00 ± 2.5 min, just before new food (5 mL of the 30% saccharose solution in a small tube, and two cut* T. molitor* mealworms for each colony) was delivered at *t*1 = 14:00 (+2.5 min). Food was maintained on the site until *t*1 + 15 min and removed at 14:15. The ants were then counted 10 times during five minutes at *t*1 + 20 min, *t*1 + 40 min, *t*1 + 60 min, and *t*1 + 80 min.

The following day (day 2), the ants were counted in the same way at the same five experimental times, but food was delivered at *t*2 = *t*1 + 20 min and removed 15 min later. At day 3, the same counting was performed and food was this time delivered at *t*3 = *t*1 + 40 min, the counting and the food removal processed as before. Similarly, at day 4, food was delivered at *t*4 = *t*1 + 60 min and the ants were counted during the same five time periods. Finally, at day 5, the ants were counted once more as before, and they received food at *t*5 = *t*1 + 80 min, during 15 minutes. One hour later, the experiment being ended, the ants were again fed* at libitum*.

The last of the 10 counts of each of the 5-minute counting intervals corresponded to the time of a possible food delivery. This 5-minute counting interval was the best one to bring to the fore expectative behavior, if it exists, by counting the ants present on the food site a little before the time of the food delivery and also the ants expecting food delivery and staying in the surrounding of the food site or watching the food delivery at a nest entrance and coming at that time. The counting time interval did of course not avoid counting a few ants randomly walking on the site but avoided counting ants coming later on after having perceived the food from some distance and those that could be recruited several minutes later onto the food. Note also that, by choosing the experimental time segment (14:00–15:35), we did not interfere with the time periods at which the species usually search for food, that is, at morning and evening.

During the first control, we counted also the ants at a subsequent time “*t*6” and saw that they were hungry, running all around their area. Also, during the experiment, each time we removed the food after the 15 experimental feeding minutes, the ants obviously wanted to go on eating and were sometimes somewhat aggressive. As a matter of fact, during the beginning of the experiments, they did not eat enough; then, since the third day, they managed to eat more, probably just sufficiently. We feared that if the experiment continued in this way, the ants would have no longer taken care of their brood and even eaten the eggs. It is the reason why we planned to end the experiment after five experimental days.

### 2.3. Statistical Analysis

**Figure 2:**
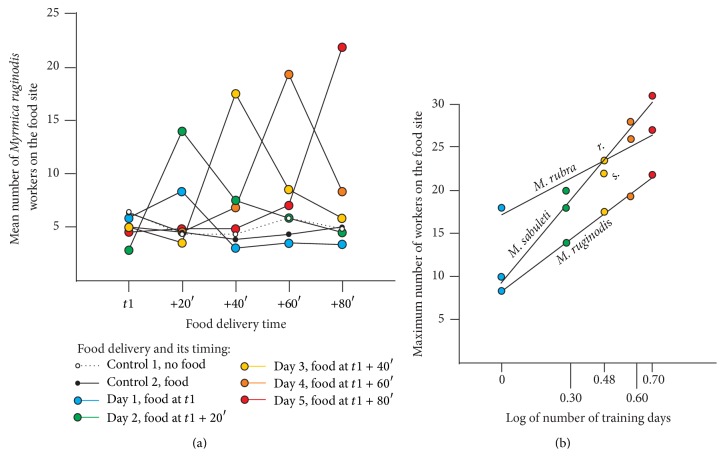
(a) Mean number of* Myrmica ruginodis* (*y*-axis) counted 10 times on the food site, at five successive potential food delivery times (*x*-axis), the effective food delivery time being delayed for 20 minutes each day. After two training days, the ants were more numerous in coming on the site at the expected time. (b) Regression lines of the maximum number of ants present each day on the food site (*y*-axis) in function of the logarithm of the number of training days (*x*-axis). The maximum number of ants increased linearly with these logarithms.

For each of the tested colonies, the means of the sums of the numbers of counted ants were compared to the values expected if ants randomly foraged on their food site in the course of time (i.e., during the five experimental time periods) using the nonparametric goodness of fit *χ* ^2^ test [13]. The five means obtained at each experimental day were also compared to one another using the nonparametric *χ* ^2^ test (same reference as above), to test if ants changed the timing of their foraging activity in the course of the daily shifting of the food delivery time. The values of *χ* ^2^, df, and *P* are given in Section 3. The relation between the maximum mean number of ants of each species on the food site and the logarithm of the number of training days (Figure 2(b)) was analyzed by their regression lines, correlation values, and Pearson's test associated probability using statistica v.10 software. The differences between the slopes and between the elevations of the obtained regression lines were compared as explained in [14].

## 3. Results

### 3.1. Concerning* Myrmica ruginodis*

#### 3.1.1. Controls

When ants had no food on their foraging area (control 1), their foraging behavior was statistically uniform in the course of time. The numbers of ants coming onto the food site during the five counting time periods did not statistically differ from those expected if ants went on that place at any time (Table 1, *χ* ^2^ = 0.66, df = 4, NS). While ants had food since day one on their food site (control 2), they also visited the site randomly according to the five successive counting times (Table 1, *χ* ^2^ = 0.76, df = 4, NS).

#### 3.1.2. Day 1

The ants were not numerous in coming on the food site at the food delivery time (*t*1), but they stayed on the site for several minutes after food was removed (Table 1). They obviously wanted to go on eating. Perceiving that food was no longer available, they finally foraged as usual and became thus again not numerous on the food site. Their presence on the site in the course of the five counting time periods did not yet differ from that expected if they came there randomly in the course of time (*χ* ^2^ = 3.97, df = 4, NS).

#### 3.1.3. Day 2

At 14:00, there were few ants on the food site. A little later, ants came out of their nest, foraged, and stopped in the vicinity of the food site. They then stayed there motionless, looking to the food site or moving in its surroundings (Figure 1(b)). At 14:20, when food was given, numerous ants quickly came onto the food site (Table 1). These ants were those waiting for food and those randomly foraging. They stayed there a few minutes after food removal, appearing somewhat nervous and aggressive, but they finally stopped looking for food and foraged as usual. The distribution of their presence on the food site at the five experimental counting time periods was now slightly different from that expected if they foraged on the site randomly in the course of time (*χ* ^2^ = 10.51, df = 4, 0.02 < *P* < 0.05).

#### 3.1.4. Days 3 and 4

At day 3, at about 14:30, several ants came out of the nest, foraged, and stayed in the vicinity of the food site, either motionless or moving sinuously. At the time of food delivery (14:40), the ants were rapidly numerous in finding the food (Table 1), and they really ate a lot (Figure 1(c)). When food was removed, they went on looking for food during a few minutes, reacted again with some aggressiveness, and then rather soon foraged as usual. Their presence on the food site was statistically different from that expected if they were there randomly in the course of time (*χ* ^2^ = 15.58, df = 4, 0.001 < *P* < 0.01). The following day, the same events happened, occurring simply 20 minutes later (Table 1). The ants did not come in large numbers on the food site until the expected food delivery time but were numerous in foraging, staying motionless or moving sinuously in its vicinity several minutes before that delivery. When food was deposited, numerous ants came on it and ate immediately, as much as they could. The timely distribution of the ants on the food site was once more statistically different from that expected if they visited randomly the site in the course of time (*χ* ^2^ = 15.11, df = 4, 0.001 < *P* < 0.01). When food was retrieved, ants reacted, trying to keep the food (Figure 1(d)), and then left the site and foraged or went back inside their nest.

#### 3.1.5. Day 5

On the fifth experimental day, the same events occurred and were more pronounced, especially the presence of ants on the food site a little before the delivery time, with postures and movement obviously revealing food expectation. When food was given, ants were soon numerous in finding and eating it, the sugar water as well as the* T. molitor *larvae (Table 1). When food was retrieved, the ants reacted but to a lower extent than previously, as if they had learned that, from now on, food was present only for a short time. The distribution of the ants' presence on the food site in the course of time was highly different from that expected if ants visited the site randomly in the course of time (*χ* ^2^ = 58.92, df = 4, *P* < 0.001). After the experiment, as stated in the experimental protocol section, ants were fed as usual.

#### 3.1.6. The Entire Experiment

The ants learned that, from now on, food would be available for only 15 minutes. The maximum number of ants counted on the food site increased thus in the course of the five experimental days (Figure 2(a)). The curve of these maxima tended to its asymptote at day 5 and appeared to be a logarithmic function of the ants' experienced shifts of the food delivery time. The pooled maximum mean number of ants of the four colonies on the food site at the food delivery time (i.e., 8.3, 14.0, 17.5, 19.3, and 21.8) is indeed a function of the logarithm of the number of training days (0, 0.3, 0.47, 0.60, and 0.69) (Figure 2(b)). More precisely, the function is as follows:

 Maximum number of ants = 19.16 lg (*t*) + 8.28, with *r* = 0.99, *r* ^2^ = 0.98, and *P* = 0.00008

Learning is thus a logarithmic function of the training time or events.

The ants also learned, day after day, to come at a later time. Indeed, their foraging distribution in the course of time statistically differed from one day to the following one, except from day 1 to day 2 (*χ* ^2^ = 3.37, df = 4, NS). At day 1, the ants stayed on the food site sometimes after food removal, thus somewhat during the time corresponding to the food delivery at day 2. The ants had then experienced only one training day. After that they came on the food site at about the subsequent correct time and stayed there more or less during the 15 feeding minutes. Their distribution among the five counting time periods differed from one day to the next one. The difference between day 3 and day 2 was already significant (*χ* ^2^ = 11.13, df = 4, 0.02 < *P* < 0.05): the ants learned the subsequent food delivery time in only two training days. Such a significant difference persisted until the end of the experiment: day 4* versus* day 3: *χ* ^2^ = 9.35, df = 4, 0.02 < *P* < 0.05; day 5* versus *day 4: *χ* ^2^ = 12.13, df = 4, 0.01 < *P* < 0.02.

### 3.2. Experiments with* M. rubra* and* M. sabuleti*

#### 3.2.1. Controls and Days 1 to 5

Numerical results are presented in Table 2.

Concerning* M. rubra, *the ants present on the food site in the absence of food were randomly distributed in the course of time (*χ* ^2^ = 3.28, df = 4, NS) though, at *t*5, a lot of ants were foraging, looking for food. In the presence of food, the ant foraging distribution was even more uniform in the time: *χ* ^2^ = 0.44, df = 4, NS. Then, in the course of the experiment, the occurrence of ants on the food site along time was not uniform. At day 1, this was not yet very significant (*χ* ^2^ = 10.03, df = 4, 0.02 < *P* < 0.05). At day 2, the ants foraged all around before the food delivery time; at about the food delivery time of day 1, they came rapidly on the food site when food was provided there and stayed there essentially during the feeding expected time (*χ* ^2^ = 15.74, df = 4, 0.001 < *P* < 0.01). This was a little more pronounced at day 3 (*χ* ^2^ = 16.63, df = 4, 0.001 < *P* < 0.01), much more at day 4 (*χ* ^2^ = 21.37, df = 4, *P* < 0.001), and largely so at day 5 (*χ* ^2^ = 40.40, df = 4, *P* < 0.001), the ants becoming then very numerous at the correct expected time, and less numerous during the other time periods. Obviously, the ants progressively waited for food a little before the expected food delivery time, doing so also from their nest entrance.

Concerning* M. sabuleti*, few ants foraged in the absence of food and their distribution in the course of time was random (*χ* ^2^ = 1.27, df = 4, NS). In presence of food, the ants were more numerous but still randomly distributed in the course of time (*χ* ^2^ = 1.66, df = 4, NS). At day 1, their distribution was still random (*χ* ^2^ = 3.96, df = 4, NS). After that, the distribution of foraging ants in the course of time became no longer random. At day 2, the ants were staying in the vicinity of the site before the delivery and could quickly come on it at the delivery time (*χ* ^2^ = 15.28, df = 4, 0.001 < *P* < 0.01). At days 3, 4, and 5, the ants came on the food site at the subsequent expected time and did not stay there at other time periods (*χ* ^2^ = 34.65, 36.41, and 54.53, resp., df = 4, *P* < 0.001). They obviously waited for food, even staying at their nest entrance (Figure 1(e)). The ants also learned to eat as much as they could during the short food delivery time, that is, sugar water, as well as meat (Figure 1(f)).

#### 3.2.2. The Entire Experiment

For pointing out the fact that the ants learned to eat a lot of during the current feeding time and to come at the correct expected time on the food site, the maximum mean numbers of ants counted on the food site were examined, and the ants' foraging distributions among the five feeding time periods observed at days 1 to 5 were compared to one another.

The maximum mean numbers of* M. rubra *and* M. sabuleti* workers counted on the food site increased in the course of the five experimental days. The curve of these maxima tended to its asymptote at day 5 and appeared to be a logarithmic function of the number of training days. The function was, for* M. rubra, as follows*:

 Maximum number of ants = 13.75 lg (*t*) + 17.24, with *r* = 0.97, *r* ^2^ = 0.94, and *P* = 0.0045

and for* M. sabuleti as follows*:

 Maximum number of ants = 30.25 lg (*t*) + 9.34, with *r* = 0.99, *r* ^2^ = 0.98, and *P* = 0.0007

These regression lines, as well as those of* M. ruginodis* (Figure 2(b)), give a good account of the learning performance of the three studied species in the course of time. However, the difference of learning speed (revealed by the slope of the regression lines) between the three species was not statistically significant (*P* > 0.50). There was also no significant difference between the elevation of the regression lines (*P* > 0.50) of* M. rubra* and* M. ruginodis*.

For* M. rubra*, the foraging distribution among the five experimental feeding times observed between day 1 and day 2 did not differ, the ants having only experienced one food delivery time shifting (*χ* ^2^ = 4.65, df = 4, NS). Thereafter, the ants' foraging distribution differed from one day to the next. The statistical results were as follows: day 3* versus *day 2: *χ* ^2^ = 18.32, df = 4, *P* < 0.001, the ants having thus learned the food delivery time shifting as soon as after having experienced two shift training; day 4* versus* day 3: *χ* ^2^ = 13.22, df = 4, *P* ~ 0.01; day 5* versus* day 4: *χ* ^2^ = 21.05, df = 4, *P* < 0.001. Similarly to* M. rubra*, the difference of the foraging distribution of* M. sabuleti *among the five feeding times was not statistically significant between day 1 and day 2 (*χ* ^2^ = 4.96, df = 4, NS), the ants having just experienced one food delivery time shift. After a second shift, the ants had learned: their foraging distribution among the five food delivery times differed from day 2 to day 3 (*χ* ^2^ = 14.23, df = 4, 0.001 < *P* < 0.01). Such a difference became highly significant between days 3 and 4 (*χ* ^2^ = 31.11, df = 4, *P* < 0.001) and between days 4 and 5 (*χ* ^2^ = 20.42, df = 4, *P* < 0.001).

## 4. Discussion

We aimed to examine if ants could expect the time at which an event (the presence of food) will occur on the basis of the previous times at which this event has occurred. We first worked on* M. ruginodis* giving food to the ants only during 15 min, always at the same place but each day delayed for 20 minutes. We observed that ants, right after two days of training, moved onto the food site at the correct expected time and progressively stayed there only during the feeding time. They were more and more numerous at the subsequent feeding time, the correlation between the maximum number of ants on the food site and the logarithm of the number of training days being highly significant. The increase was thus a logarithmic function of the number of training days. We made the same experiment on two colonies of* M. rubra *and* M. sabuleti* and observed the same behavior; that is, during the first experimental days, the ants presented some aggressiveness when food was retrieved, and progressively, they more and more correctly wait for food some time before the expected subsequent delivery time. These ants' learning also appeared to be a highly significant logarithmic function of the number of training days, and the ants' foraging distribution among the five food delivery periods varied significantly each day from day 2 onwards.

Ants learned quickly to expect food at the correct time probably because they primarily learned that food stayed available for only 15 minutes and that they had to eat as much as possible within this time. The food removal after 15 minutes could have been perceived as a negative reward (a punishment). A negative reward leads to a quick acquisition of conditioning, though not to a better score than conditioning with a reward (e.g., [15]). Consequently, the ants adapted themselves rather quickly to the situation: they came in the vicinity of the food site and inspected it at the correct time; and they collected as much food as they could within the 15 feeding minutes. The fact that removing food acted as a “punishment” was pointed out by the ants' reaction during such a removal: they were aggressive, defended the food, and tried to keep it, but essentially during the first days of the experiment. After the end of the experiment, when ants were again fed* ad libitum*, they were not numerous in coming on the provided food, being thus not very hungry: they could adapt themselves to the experimental feeding planning (each day, food delivered 20 min later, each time for 15 min).

No statistical difference was found between the slopes and elevations of the regression lines accounting for the learning performance of the three experimented species. This may be due to the small number of training days used in the present experimental work. Other experiments should be undertaken for looking to a potential difference of cognitive performance between different species and even between several colonies belonging to the same species. Indeed, working on* Aphaenogaster senilis*, Blight et al. demonstrated that, under standardized laboratory conditions, different colonies of the same age do have different personalities and vary in their exploration, risk taking, food retrieval, and nestmates interactions [16].

Such an expectative behavior about the time of an event occurrence required several ethological and physiological abilities: estimating the time running, knowing the environment and the place where food should be present, being prevalent, having memory, and being able to act anticipatively.* Myrmica *ants have these abilities. It is thus not surprising that these insects could acquire some expectation concerning the time of occurrence of an event. Although not all ant species, insects, or other animals may detain such ability, several ones could have it since it is advantageous in nature. This is the case for* M. ruginodis*,* M. sabuleti*, and* M. rubra*: these ants collect honeydew from aphids as well as floral nectar, the occurrence time of which may vary day after day and/or may be available only for a short time. Being able to collect food efficiently during the short time of its availability and looking for food only at about the time of its expected presence should allow efficient food collection and energy sparing. Experiments similar to the present one should be tempted on other animal species such as bumblebees, bees, rats, monkeys, and human children of different ages. Concerning bumblebees, a kind of expectation has been observed. These insects check the quality and quantity of the flower nectar (they take some nectar from one flower) before foraging on a new patch of flowers and go on foraging only if the harvest is expected to be higher than a given threshold under which their energy intake would not be optimal [17, 18]. Further studies on these hymenoptera should be performed, looking this time for potential expectative behavior about the time of food occurrence. Bees have an excellent notion of the running time; they collect nectar, a food source not always available. Experimenting on such insects should be of interest, more so since honeybees have been shown to exhibit some expectation as for being rewarded [19]. At first sight, rodents do not need to expect the time at which food will be available. It would be interesting to see if, under artificial experimental conditions, such mammals could nevertheless acquire expectation as for the time of an event occurrence. Recent neuronal studies are in favor of some expectation ability in rats, at least about the fact of being rewarded [20]. Such kind of expectation has also been observed in monkeys [21]. So, experimenting, like in the present work, on these mammals should provide information on the subject. Finally, experimenting on monkeys and on human children of different ages should inform about the ontogenesis of the expectative behavior here examined. Let us recall that expectation for the location of food is not innate in ants but acquired rather later during their life experience [7]. Anyway, having the ability to expect the change in location and/or in time of an event provides an obvious advantage to the animals.

## 5. Conclusion

The present work shows that, right after having experienced two feeding times lasting only 15 minutes and two shifts of a 20-minute delay in the feeding time, ants of three* Myrmica* species could adapt themselves to the situation, that is, eating as much as possible within 15 minutes and coming on the food site at the subsequent expected time. Such an expectative behavior is presumed to exist in other sufficiently evolved animal species and is of course an advantageous ethological trait.

## Figures and Tables

**Table 1:** Number of ants of four colonies of *Myrmica ruginodis* counted on the food site at five successive times during five consecutive days, the food being delivered during 15 min at a given time “*t*” which was delayed for 20 min each day.

Days, food delivery times, and counting times (*t*1–*t*5)	Colonies				Mean
	A	B	C	D	
Control 1, no food					
*t*1	0	15	5	5	6.3
*t*2 = *t*1 + 20′	1	0	10	6	4.3
*t*3 = *t*1 + 40′	3	2	7	5	4.3
*t*4 = *t*1 + 60′	8	2	3	10	5.8
*t*5 = *t*1 + 80′	4	4	4	7	4.8

Control 2, food present					
*t*1	10	0	5	10	6.3
*t*2 = *t*1 + 20′	10	0	5	3	4.5
*t*3 = *t*1 + 40′	5	0	5	5	3.8
*t*4 = *t*1 + 60′	5	4	5	3	4.3
*t*5 = *t*1 + 80′	10	0	5	5	5.0

Day 1, food given at *t*1 and retrieved at the end of *t*1					
**t**1	10	7	6	0	**5.8**
*t*2 = *t*1 + 20′	2	4	22	5	**8.3**
*t*3 = *t*1 + 40′	0	2	3	7	3.0
*t*4 = *t*1 + 60′	2	7	5	0	3.5
*t*5 = *t*1 + 80′	1	4	7	1	3.3

Day 2, food given at *t*2 and retrieved at the end of *t*2					
*t*1	5	0	4	2	2.8
**t**2 = *t*1 + 20′	11	13	26	6	**14.0**
*t*3 = *t*1 + 40′	5	9	8	8	7.5
*t*4 = *t*1 + 60′	0	13	10	0	5.8
*t*5 = *t*1 + 80′	0	2	11	5	4.5

Day 3, food given at *t*3 and retrieved at the end of *t*3					
*t*1	0	9	3	8	5.0
*t*2 = *t*1 + 20′	3	4	2	5	3.5
**t**3 = *t*1 + 40′	15	11	31	13	**17.5**
*t*4 = *t*1 + 60′	10	7	9	8	8.5
*t*5 = *t*1 + 80′	2	7	10	4	5.8

Day 4, food given at *t*4 and retrieved at the end of *t*4					
*t*1	1	7	7	5	5.0
*t*2 = *t*1 + 20′	6	4	5	2	4.5
*t*3 = *t*1 + 40′	1	9	11	6	6.8
**t**4 = *t*1 + 60′	18	14	18	27	**19.3**
*t*5 = *t*1 + 80′	1	10	4	18	8.3

Day 5, food given at *t*5 and retrieved at the end of *t*5					
*t*1	7	0	8	3	4.5
*t*2 = *t*1 + 20′	5	2	6	6	4.8
*t*3 = *t*1 + 40′	1	7	9	2	4.8
*t*4 = *t*1 + 60′	6	3	7	12	7.0
**t**5 = *t*1 + 80′	15	18	27	27	**21.8**

**Table 2:** Same legend as for Table 1, except that the experiment was made on two colonies of *M. rubra *and two colonies of *M. sabuleti*.

Days, food delivery times, and counting times (*t*1–*t*5)	*M. rubra* colonies		Mean	*M. sabuleti* colonies		Mean
	A	B		A	B	
Control 1, no food						
*t*1	10	5	7.5	2	0	1.0
*t*2 = *t*1 + 20′	8	5	6.5	4	2	3.0
*t*3 = *t*1 + 40′	8	0	4.0	2	2	2.0
*t*4 = *t*1 + 60′	10	0	5.0	2	2	2.0
*t*5 = *t*1 + 80′	20	0	10.0	3	3	3.0

Control 2, food present						
*t*1	26	10	18.0	10	5	7.5
*t*2 = *t*1 + 20′	20	10	15.0	10	5	7.5
*t*3 = *t*1 + 40′	20	10	15.0	10	10	10.0
*t*4 = *t*1 + 60′	20	10	15.0	10	5	7.5
*t*5 = *t*1 + 80′	20	10	15.0	0	10	5.0

Day 1, food given at *t*1 and retrieved at the end of *t*1						
**t**1	17	6	**11.5**	15	5	**10.0**
*t*2 = *t*1 + 20′	30	6	**18.0**	10	5	**7.5**
*t*3 = *t*1 + 40′	18	3	**10.5**	7	12	**9.5**
*t*4 = *t*1 + 60′	10	3	6.5	8	0	4.0
*t*5 = *t*1 + 80′	10	0	5.0	0	10	5.0

Day 2, food given at *t*2 and retrieved at the end of *t*2						
*t*1	9	0	4.5	5	6	6.5
**t**2 = *t*1 + 20′	24	16	**20.0**	21	15	**18.0**
*t*3 = *t*1 + 40′	11	0	10.5	8	8	8.0
*t*4 = *t*1 + 60′	10	0	5.0	5	5	5.0
*t*5 = *t*1 + 80′	11	8	9.5	0	8	4.0

Day 3, food given at *t*3 and retrieved at the end of *t*3						
*t*1	10	10	10.0	0	10	5.0
*t*2 = *t*1 + 20′	3	11	7.0	7	4	5.5
**t**3 = *t*1 + 40′	33	14	**23.5**	24	20	**22.0**
*t*4 = *t*1 + 60′	15	13	14.0	3	3	3.0
*t*5 = *t*1 + 80′	10	2	6.0	3	2	2.5

Day 4, food given at *t*4 and retrieved at the end of *t*4						
*t*1	18	0	9.0	4	3	3.5
*t*2 = *t*1 + 20′	4	10	7.0	5	3	4.0
*t*3 = *t*1 + 40′	9	6	7.5	8	8	8.0
**t**4 = *t*1 + 60′	30	22	**26.0**	22	34	**28.0**
*t*5 = *t*1 + 80′	10	10	10.0	10	14	12.0

Day 5, food given at *t*5 and retrieved at the end of *t*5						
*t*1	9	0	4.5	7	2	4.5
*t*2 = *t*1 + 20′	4	4	4.0	5	0	2.5
*t*3 = *t*1 + 40′	7	4	5.5	6	4	5.0
*t*4 = *t*1 + 60′	8	6	7.0	8	8	8.0
**t**5 = *t*1 + 80′	34	20	**27.0**	34	28	**31.0**
